# *Level Set method*-based two-dimensional numerical model for simulation of nonuniform open-channel flow

**DOI:** 10.1371/journal.pone.0223167

**Published:** 2019-09-26

**Authors:** Rui Xu, Shihe Liu

**Affiliations:** State Key Laboratory of Water Resources and Hydropower Engineering Science, Wuhan University, Wuhan, P. R. China; China University of Mining and Technology, CHINA

## Abstract

The capture precision of the free surface of an open-channel with a water-air interface directly affects the calculation precision of flow field characteristics and general characteristics of the flow. Significant research effort has been devoted to Level Set since its creation, although the relevant research is mainly limited to bubble or droplet movement. In this paper, Level Set method is applied to a two-dimensional numerical simulation of open-channel turbulence, while a new numerical model is proposed and multispot synchronized experimental data are applied to the validation of numerical model. In addition, the model is used to study the flow field characteristics and general characteristics of open-channel flow, which have a water-level lowering curve. The study shows that (1) a semilogarithm zone of vertical distribution of longitudinal velocity is still present amid the transition of flow from nonuniform to uniform, and the depth-averaged velocity and wall shear stress increase along the flowing path. (2) both the energy loss coefficient and roughness coefficient of the flow at nonuniform flow region are greater than the respective values at uniform flow region, and the magnitude of the deviation is relevant to the magnitude of the flow deviation from uniform flow stage.

## Introduction

The flow in natural river course and artificial open-channel belongs to open-channel flow, which is characterized by the existence of free surface (water-air interface) [1,2]. Both the flood water-level in water resources and lowest navigable water-level in navigation are characteristic quantity representing free surface position. They are important parameters for measuring flood discharge capacity and navigation capacity of rivers in reality[3,4].

Research regarding open-channel flow through experimental research and numerical calculations has been completed by many researchers. In terms of experimental research, Nezu and Sanjou [5] adopted a laser Doppler anemometer (LDA) to study uniform open-channel flow turbulence, concluding that only near-wall flow velocity could be described using a logarithmic law, whereas a systematic deviation occurs between the far-wall mainstream flow velocity distribution that can be described using the logarithmic law and Coles wake flow law; T. Song and Y. M. Chiew [6] conducted a nonuniform flow experiment in a long water channel to study the impact of nonuniform flow on water movement and concluded that there is a relationship between the distribution of the parameters such as the flow velocity and turbulence intensity and the uniformity of the flow; A. H. Cardoso et al. [7] studied the distribution law of flow velocity and wall shear stress in accelerated flow. In terms of numerical calculations, K. Shiono et al. [8] studied flow in prism open-channel with complicated cross-section and deduced a depth-averaged velocity formula and wall shear stress transverse distribution equation; Arun Kamath et al. [9] established a numerical model based on the Reynolds Averaged Navier-Stokes (RANS) equations to simulate the complex surface flows over weirs around the bridge piers; Krishna Chandran et al. [10]studied the dam break flow, two-dimensional cavity filling, etc. by the volume of fluid (VOF) method to demonstrate the validity of the surface flow model; A. Khosronejad et al. [11] carried out the study of open-channel flow with a wall-mounted bridge abutment and compared the numerical simulation results of rigid-lid and level set method.

Three core issues exist in numerical simulation of open-channel turbulence, i.e., the choice of the turbulence model, discretization of the governing equation and treatment of the free surface [12]. A good turbulence model is particularly important for complicated boundary flow. But for a flow with simple boundary, such as the flow in a long straight open-channel, even the common *k*-*ε* model can basically reflect its average flow field characteristics. For the discretization of the governing equation, the finite difference method (FDM) or finite control volume is currently used, and many research results are available with relatively mature study methods.

In relative terms, the free surface treatment of open-channel flow remains to be improved in numerical simulations [13,14]. Rigid-lid and VOF are the two common methods of water surface handling at present [15,16,17]. The former is adopted to address a free surface problem of steady uniform-flow, but some researchers [11] pointed out that the reliable results may not be obtained by rigid-lid under certain conditions. The latter is used in mainstream business computing software for performing computations of the free surface of a nonuniform flow [18,19]. So we’ll choose the VOF method as the comparison object for Level Set method. In comparison, Level Set has the following advantages: (1) for the Level Set method, the free surface can be directly obtained by its definition, but for the VOF, additional geometric reconstruction must be carried out, and the results are different with different reconstruction strategies [20,21]; (2) most of the free surfaces obtained by Level Set are clear and sharp, while the results obtained by VOF may be blurred and serrated[22,23]; (3) it is easy to get a complete image by Level Set method, but the using of VOF may lead to numerical crushing, resulting in scattered images[24,25].

There are also cases in which Level Set is adopted to study the free surface and the formation of bubbles in water and during the disengagement process [24,26,27], and it has been successfully used in numerical simulations of general free surface flow[28,29,30]; however, there are few numerical studies regarding open-channel turbulence that adopt Level Set. Moreover, it is particularly important to analyze parameters such as wall shear stress, roughness coefficient and mechanical energy loss coefficient while studying the open-channel flow evolution from nonuniform flow to uniform flow. Relevant research has not been extensively conducted due to the limit of many conditions.

Therefore, on the basis of the existing research work, this paper has carried out the following original work: (1) Level Set method was recommended to capture the free surface of open-channel flow; (2) a vertical two-dimensional numerical model based on Level Set method was established; (3) an experiment of open channel flow was carried out in a long straight tank, and the experimental results were obtained by, which were employed to test the numerical model; (4) the numerical results were used to study the variation tendency of hydraulic characteristics, during the transition of open-channel flow from non-uniform to uniform.

It’s worth noting that there are many different types of flows in the open-channel flow, such as backwater flow, dropdown flow, critical flow, etc. We chose one of the most typical flow, which have a water-level lowering curve, as the research object according to the experimental conditions.

## Mathematical model

### Free surface equation

Level Set was first proposed by Osher and others in 1988 [20]. The basic idea behind Level Set is to translate m-dimensional curve plane motion evolution to m+1-dimensional function projection and implicitly solve the high-dimensional function, thus precisely describing the low-dimensional curve plane topological structure.

**Fig 1:**
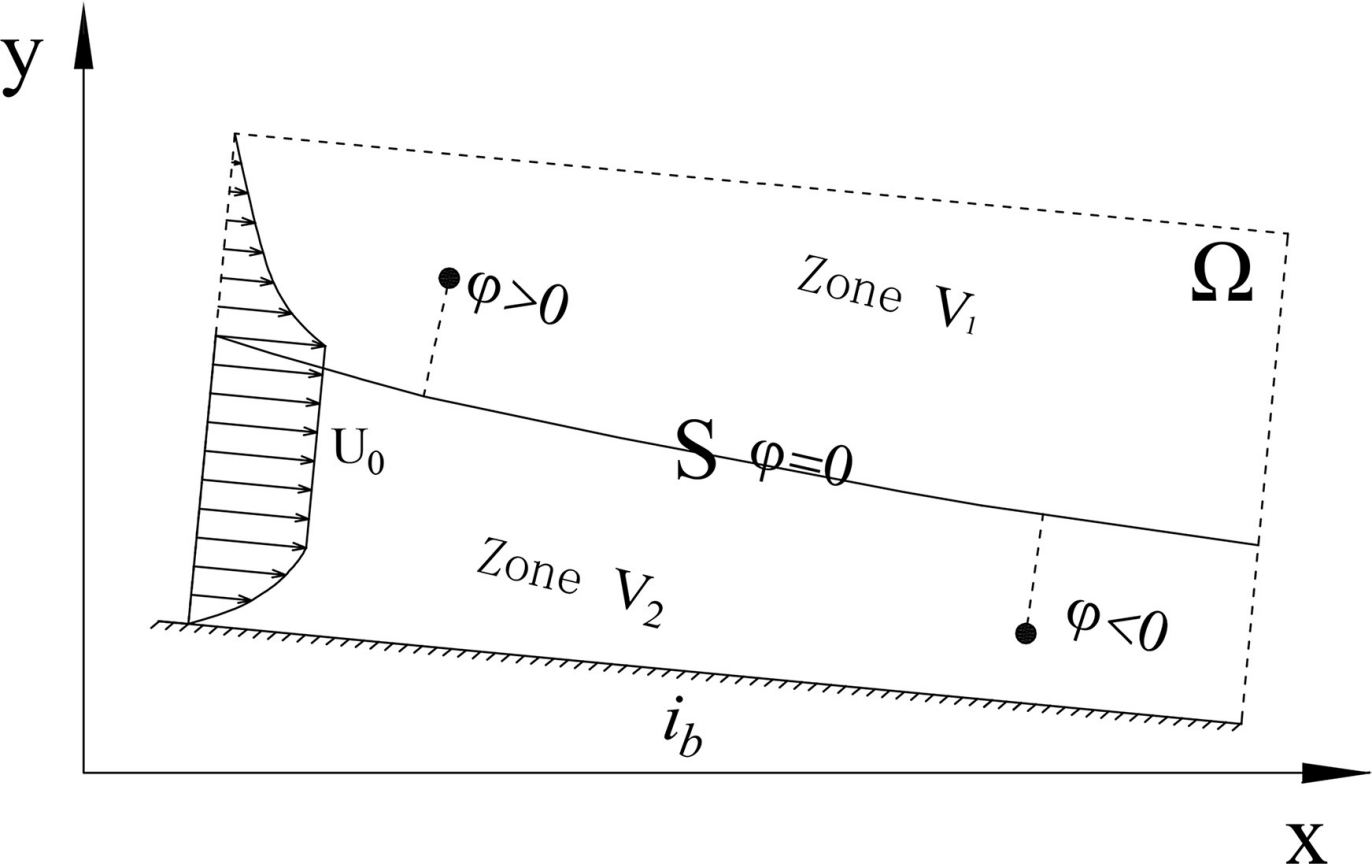
Schematic diagram of free surface flow problem. The whole domain *Ω* was divided into three parts: zone *V*_1_, zone *V*_2_ and interface *S*. *φ* is the symbolic distant function.

As shown in Fig 1 above, the open-channel profile of the two-dimensional flow is zoned airflow *V*_1_, water flow *V*_2_ and interface *S*(*x*,*t*) between *V*_1_ and *V*_2_. The constructor *φ*(*x*,*y*,*t*) makes the free surface *S*(*x*,*t*) the exact zero isoline of function *φ*(*x*,*y*,*t*) at any moment, and the starter of *φ*(*x*,*y*,*t*) meets the conditions for normal direction monotony in the vicinity of free surface *S*(*x*, *t*). It is apparent that the requirements can be met when *φ*(*x*,*y*,*t*) is taken as the symbolic distance between point (*x*,*y*) and interface *S*(*x*,*t*), i.e.,

$$\varphi (x,y,t)=)\left\{ \begin{array}{l} d((x,y),S(x,t)),\;(x,y)\in V_{1}\\ \;0,\;(x,y)\in S\\ -d((x,y),S(x,t)),\;(x,y)\in V_{2} \end{array}\right.$$ (1)

where *d*(*A*,*B*) is the distance of *A* and *B*, *S*(*x*,*t*) is the interface of fluid, *φ* is the symbolic distant function.

There is always *φ*(*x*, *y*, *t*) = 0 at any moment for a point on the free surface, leading to $\frac{d\varphi }{dt}=0$. The following equation is obtained thereafter:

$$\frac{\partial \varphi }{\partial t}+u\frac{\partial \varphi }{\partial x}+v\frac{\partial \varphi }{\partial y}=0$$ (2)

where *u* and *v* are the RANS flow velocity in the *x* and *y* directions, respectively. Eq (2) is called the Level Set equation, in which the symbolic distant function *φ*(*x*, *y*, *t*) is the Level Set function.

### Motion equation

The calculation zones *Ω* consist of airflow zone *V*_1_ and water flow zone *V*_2_. The RANS equation is used to describe the variation in the amount of the RANS flow characteristic in the main zones, and the standard *k*-*ε* model is used to compute the Reynolds stress. The corresponding equations are as follows:

Continuity equation:

$$\frac{\partial u}{\partial x}+\frac{\partial v}{\partial y}=0$$ (3)

Momentum equations:

$$\frac{\partial \rho u}{\partial t}+\frac{\partial \rho uu}{\partial x}+\frac{\partial \rho uv}{\partial y}=\rho g\sin\theta -\frac{\partial p}{\partial x}+\frac{\partial }{\partial x}\left(2\mu \frac{\partial u}{\partial x}\right)+\frac{\partial }{\partial y}\left[\mu \left(\frac{\partial u}{\partial y}+\frac{\partial v}{\partial x}\right)\right]+\frac{\partial }{\partial x}\left(-\rho \bar{u^{'}u^{'}}\right)+\frac{\partial }{\partial y}\left(-\rho \bar{u^{'}v^{'}}\right)$$ (4)

$$\frac{\partial \rho v}{\partial t}+\frac{\partial \rho uv}{\partial x}+\frac{\partial \rho vv}{\partial y}=\rho g\cos\theta -\frac{\partial p}{\partial y}+\frac{\partial }{\partial x}\left[\mu \left(\frac{\partial u}{\partial y}+\frac{\partial v}{\partial x}\right)\right]+\frac{\partial }{\partial y}\left(2\mu \frac{\partial v}{\partial y}\right)+\frac{\partial }{\partial x}\left(-\rho \bar{u^{'}v^{'}}\right)+\frac{\partial }{\partial y}\left(-\rho \bar{v^{'}v^{'}}\right)$$ (5)

Turbulent kinetic energy (*k*) equation:

$$\frac{\partial \rho k}{\partial t}+\frac{\partial \rho uk}{\partial x}+\frac{\partial \rho vk}{\partial y}=G_{k}+\frac{\partial }{\partial x}\left[\left(\mu +\frac{\rho \nu_{t}}{\sigma_{k}}\right)\frac{\partial k}{\partial x}\right]+\frac{\partial }{\partial y}\left[\left(\mu +\frac{\rho \nu_{t}}{\sigma_{k}}\right)\frac{\partial k}{\partial y}\right]$$ (6)

Turbulent kinetic energy dissipation rate (*ε*) equation:

$$\frac{\partial \rho \varepsilon }{\partial t}+\frac{\partial \rho u\varepsilon }{\partial x}+\frac{\partial \rho v\varepsilon }{\partial y}=C_{\varepsilon 1}\rho \frac{\varepsilon }{k}G_{k}+\frac{\partial }{\partial x}\left[\left(\mu +\frac{\rho \nu_{t}}{\sigma_{\varepsilon }}\right)\frac{\partial \varepsilon }{\partial x}\right]+\frac{\partial }{\partial y}\left[\left(\mu +\frac{\rho \nu_{t}}{\sigma_{\varepsilon }}\right)\frac{\partial \varepsilon }{\partial y}\right]-C_{\varepsilon 2}\rho \frac{\varepsilon^{2}}{k}$$ (7)

where *u* and *v* are flow velocity in the *x* and *y* directions, respectively; *u*′,*v*′ are fluctuating velocity components; $-\rho \bar{u^{'}v^{'}}$ is Reynolds stress; *p* is the pressure intensity; *G*_k_ is the produced turbulent kinetic energy; *θ* is the dip angle of open-channel; *ρ* and *μ* are the density and dynamic viscosity coefficient of air and water, respectively; ν_t_ is the turbulent viscosity coefficient; *C*_*μ*_, *σ*_k_, *σ C*_ε1_ and *C*_ε2_,are all empirical coefficients, which are valued for the constant in the standard *k*-*ε* model[31]: *C*_μ_ = 0.09, *σ*_k_ = 1.00, *σ*_*ε*_ = 1.30, *C*_ε1_ = 1.44, *C*_ε2_ = 1.92(S1 Table).

## Numerical method

A numerical simulation calculation usually comprises grid division, discretization of the equation, boundary condition settings, and the equation solution. They are elaborated in this section.

The rectangular structure grid is used to divide the calculated field in the calculation.

### Discretization of the motion equation

The motion equation may be commonly expressed by the following pattern:

$$\frac{\partial \Phi }{\partial t}+u\frac{\partial \Phi }{\partial x}+v\frac{\partial \Phi }{\partial y}=\frac{\partial }{\partial x}\left(\Gamma \frac{\partial \Phi }{\partial x}\right)+\frac{\partial }{\partial y}\left(\Gamma \frac{\partial \Phi }{\partial y}\right)+S_{0}\left(\Phi \right)$$ (8)

where Φ is the generalized variable, Γ is the generalized diffusive coefficient, and *S*_0_(Φ) is the source term. These variables have different meanings in different equations.

This motion equation(RANS) is discretized by using a finite volume method(FVM)[32], and the SIMPLE algorithm[33] is used to solve the problem of pressure-velocity coupling. We use the second-order QUICK scheme for the convective terms in the RANS equation and the *k* and *ε* equations[34]. A central differencing scheme is used to discretize the viscous terms. The detailed discretization of RANS equation are given in S1 Appendix.

### Discretization of level set equation

It is not a simple task to solve the Level Set equation directly, because the Level Set function is implicit. Therefore, some efficient measures must be taken to deal with the equation. In reference [35], the Eq (2) is rewritten as the spatial derivative operator below:

$$\frac{\partial \varphi }{\partial t}=L(\varphi )=-u\frac{\partial \varphi }{\partial x}-v\frac{\partial \varphi }{\partial y}$$ (9)

where *L(φ)* is the spatial operator, *φ* is the Level Set function.

We use different schemes to discretize the left and right sides of the rewriting equation above as follows:

At the left end of the equation, third-order Runge-Kutta is used for time discretization:

$$\{\begin{array}{l} \varphi^{\left(1\right)}=\varphi^{\left(0\right)}+\Delta tL(\varphi^{\left(0\right)})\\ \varphi^{\left(2\right)}=\frac{3}{4}\varphi^{\left(0\right)}+\frac{1}{4}\varphi^{\left(1\right)}+\frac{1}{4}\Delta tL(\varphi^{\left(1\right)})\\ \varphi^{\left(3\right)}=\frac{1}{3}\varphi^{\left(0\right)}+\frac{2}{3}\varphi^{\left(2\right)}+\frac{2}{3}\Delta tL(\varphi^{\left(2\right)}) \end{array}$$ (10)

At the right end of the equation, the fifth-order WENO combined structured template is used to directly compute the values of *φ*_*x*_ and *φ*_*y*_, obtaining the *L*(*φ*) value.

**Fig 2:**
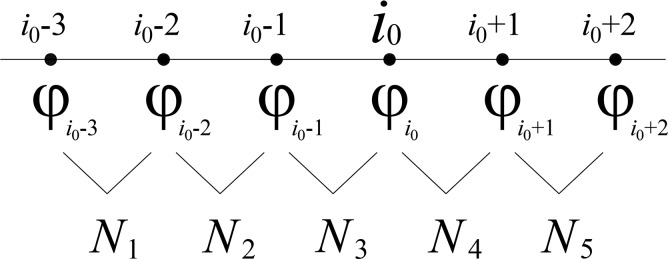
WENO node distribution. The newton difference quotients N are obtained from adjacent node values.

For example, using the *x* direction, determine two derivatives from the left end and right end for any point *i*_0_, i.e., the left derivative *φ- x* and right derivative *φ+ x*. To solve the left derivative, as shown in Fig 2, choose six node templates {$\varphi_{i_{0}-3}^{}$, $\varphi_{i_{0}-2}^{}$, $\varphi_{i_{0}-1}^{}$, $\varphi_{i_{0}}^{}$, $\varphi_{i_{0}+1}^{}$, $\varphi_{i_{0}+2}^{}$}, and obtain the five first-order Newton difference quotients, *N*_1_, *N*_2_, *N*_3_, *N*_4_, *N*_5_.

To obtain the right derivative, choose another six node templates {$\varphi_{i_{0}-2}^{}$, $\varphi_{i_{0}-1}^{}$, $\varphi_{i_{0}}^{}$, $\varphi_{i_{0}+1}^{}$, $\varphi_{i_{0}+2}^{}$, $\varphi_{i_{0}+3}^{}$}, and similarly obtain the five first-order Newton difference quotients, *N*_1_, *N*_2_, *N*_3_, *N*_4_, *N*_5_.

Following the WENO method, the convective term discretization pattern through weighting is obtained:

$${\left(\varphi_{x}^{\pm }\right)}_{i_{0}}=\omega_{1}(\frac{N_{1}}{3}-\frac{7N_{2}}{6}+\frac{11N_{3}}{6})+\omega_{2}(\frac{-N_{2}}{6}+\frac{5N_{3}}{6}+\frac{N_{4}}{3})+\omega_{3}(\frac{N_{3}}{3}+\frac{5N_{4}}{6}-\frac{N_{5}}{6})$$ (11)

where parameter *ω* is the weighting coefficient. It can be obtained it by the reference [36]:

$$\begin{array}{l} \omega_{1}=\frac{a_{1}}{a_{1}+a_{2}+a_{3}}\\ \omega_{2}=\frac{a_{2}}{a_{1}+a_{2}+a_{3}}\\ \omega_{3}=\frac{a_{3}}{a_{1}+a_{2}+a_{3}} \end{array}$$ (12)

where *a* is the transition coefficient, they are given as follows:

$$\begin{array}{l} a_{1}=\frac{0.1}{{\left(\varepsilon_{0}+IS_{1}\right)}^{2}}\\ a_{2}=\frac{0.6}{{\left(\varepsilon_{0}+IS_{2}\right)}^{2}}\\ a_{3}=\frac{0.3}{{\left(\varepsilon_{0}+IS_{3}\right)}^{2}} \end{array}$$ (13)

$$\begin{array}{l} IS_{1}=\frac{13}{12}{\left(N_{i_{0}-2}-2N_{i_{0}-1}+N_{i_{0}}\right)}^{2}+\frac{1}{4}{\left(N_{i_{0}-2}-4N_{i_{0}-1}+3N_{i_{0}}\right)}^{2}\\ IS_{2}=\frac{13}{12}{\left(N_{i_{0}-1}-2N_{i_{0}}+N_{i_{0}+1}\right)}^{2}+\frac{1}{4}{\left(N_{i_{0}-1}-N_{i_{0}+1}\right)}^{2}\\ IS_{3}=\frac{13}{12}{\left(N_{i_{0}}-2N_{i_{0}+1}+N_{i_{0}+2}\right)}^{2}+\frac{1}{4}{\left(3N_{i_{0}}-4N_{i_{0}+1}+N_{i_{0}+2}\right)}^{2} \end{array}$$ (14)

where *ε*_0_ is an extremely small number which is used to avoid the denominator of *a* becoming zero. In our later computation, the value of *ε*_0_ is 10^−7^_._

With the upwind scheme concept, *φ*_*x*_ is equal to *φ- x* when *u*_*i*0_>0 or *φ+ x* when *u*_*i*0_<0 for node *i*_0_. The calculation in the *y* direction is consistent with the *x* direction, obtaining (*φ*± *y*) in the same manner(S2 Appendix).

### Boundary conditions

As shown in Fig 1 above, the boundaries of the calculation fields include the inlet boundary, outlet boundary, pressure boundary and wall boundary.

(I) Inlet boundary

The inlet boundary gives the flow velocity at the intake and assumes a uniform distribution, i.e., *U = U*_0_, on the basis of available conditions.

(II) Outlet boundary

The pressure boundary is used as the outlet boundary. The pressure is static and therefore used as a constant. The atmospheric pressure is used as the pressure boundary in the calculation since this boundary is open to the atmosphere, i.e., *P = P*_atm_.

(III) Wall boundary conditions

There are no penetration and no slip conditions applied on the wall boundary, i.e., *u =* 0, *v =* 0. The wall function is applied in the calculation for the wall side and vicinity, and the flow velocity satisfies $u^{+}=\frac{1}{\kappa }\ln\left(Ey^{+}\right)$ and $y^{+}=\frac{\Delta y_{p}(C_{\mu }{}^{1/4}k_{p}{}^{1/2})}{\mu }$, where *u*^+^,*y*^+^ is the dimensionless parameters, Δ*y*_*p*_ is the distance of the near-wall node *P* to the solid surface, Von Karman’s constant *κ* = 0.41, wall roughness parameter *E* = 9.8. See reference [37] for the selection of the relevant parameters and calculations.

### Main numerical step

**Fig 3:**
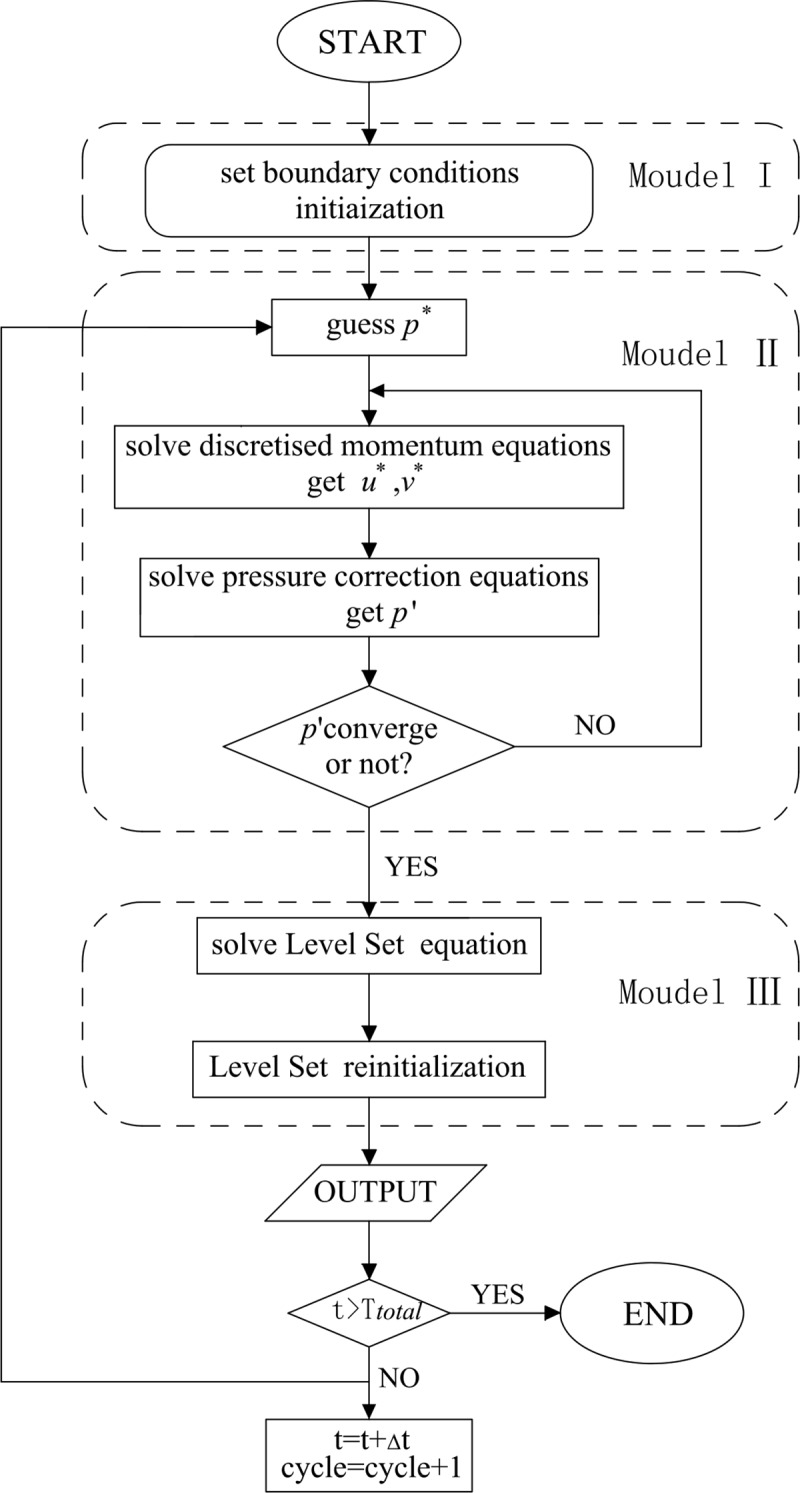
Calculation flow chart.

The principal calculation program consists of two modules, i.e., the motion equation solution module and the free surface solution module. They are mobilized for the circulation calculation after the initial assignment for the resulting output based on the condition of convergence. The calculation procedure is given in Fig 3.

- Initial assignment: set the boundary condition and assign starters for the entire field.
- Motion equation solution:
  - Solve the motion equation coefficient and motion equation.
  - Solve the *k* and *ε* equation.
  - Obtain the pressure intensity correction equation coefficient, solve the corrected value, and update the pressure intensity and flow velocity.
  - Repeat steps (b)-(d) until all parameters are converged, and then proceed to the next module.
- Free surface equation solution:
  - Obtain *φ*_t_ with reference to the obtained flow field (*u*, *v*) and last moment *φ*_t-1_.
  - Reinitialize *φ*_t_, keep the symbolic characteristics, and obtain output result.
  - Determine whether to proceed to next stage calculation or end it based on the time calculation.

### Key issue handling

The following issues are addressed in order to guarantee precision in the calculations:

(a) the water flow-air density ratio *ρ*_w_/*ρ*_a_ = 828.4 and viscosity coefficient ratio *μ*_w_/*μ*_a_ = 55.5 may destabilize the calculation given the high *ρ*-*μ* difference on both sides of the free surface. Therefore, the physical property parameters in the vicinity of the interface must be smoothly treated. Consequently, the Heaviside function *H* is defined as follows:

$$H(\varphi )=\left\{ \begin{array}{l} \;0\;(if\;\varphi <-\varepsilon_{1})\\ \left(\varphi +\varepsilon_{1})/2\varepsilon_{1}+\sin(\pi \varphi /\varepsilon_{1})/2\pi \;(if\;\left|\varphi \right|\leq \varepsilon_{1}\right)\\ \;1\;(if\;\varphi >\varepsilon_{1}) \end{array}\right.$$ (15)

where *ε*_1_ is the low amount rectifying parameter, usually *ε*_1_ = 1.5*O*(Δ*x*,Δ*y*), we take *ε*_1_ = 1.5Δ*y* in this calculation. *ρ* and *μ* in the whole field can be expressed as

$$\left\{ \begin{array}{l} \rho (x,y)=\rho_{a}+(\rho_{w}-\rho_{a})H(\varphi )\\ \mu (x,y)=\mu_{a}+(\mu_{w}-\mu_{a})H(\varphi ) \end{array}\right.$$ (16)

where the subscripts *w* and *a* represent water and air, respectively, *H*(*φ*) is smoothed Heaviside function.

(b) After a certain time increment, function *φ*(*x*, *y*) no longer meets Eq (1) defined as the symbolic distance characteristics in the numerical calculation. In this regard, *φ*(*x*, *y*) at the end of each stage is initialized to meet the requirement, a solution to the following initial value problem:

$$\left\{ \begin{array}{l} \varphi =sign(\varphi_{0})(1-\left|\nabla \varphi \right|)\\ \varphi (x,y,0)=\varphi_{0} \end{array}\right.$$ (17)

The computational details of the reinitialization are given in S3 Appendix.

## Experimental study and validation of numerical model

### Experimental equipment and methods

The experiment is conducted in a 28-m long, 0.56-m wide and 0.7-m high sloped channel. The sides and bottom of the channel are made of glass. A laser water-level gauge is used to measure the water-level, and an acoustic Doppler velocity meter (ADV) is used to measure the flow velocity.

**Fig 4:**
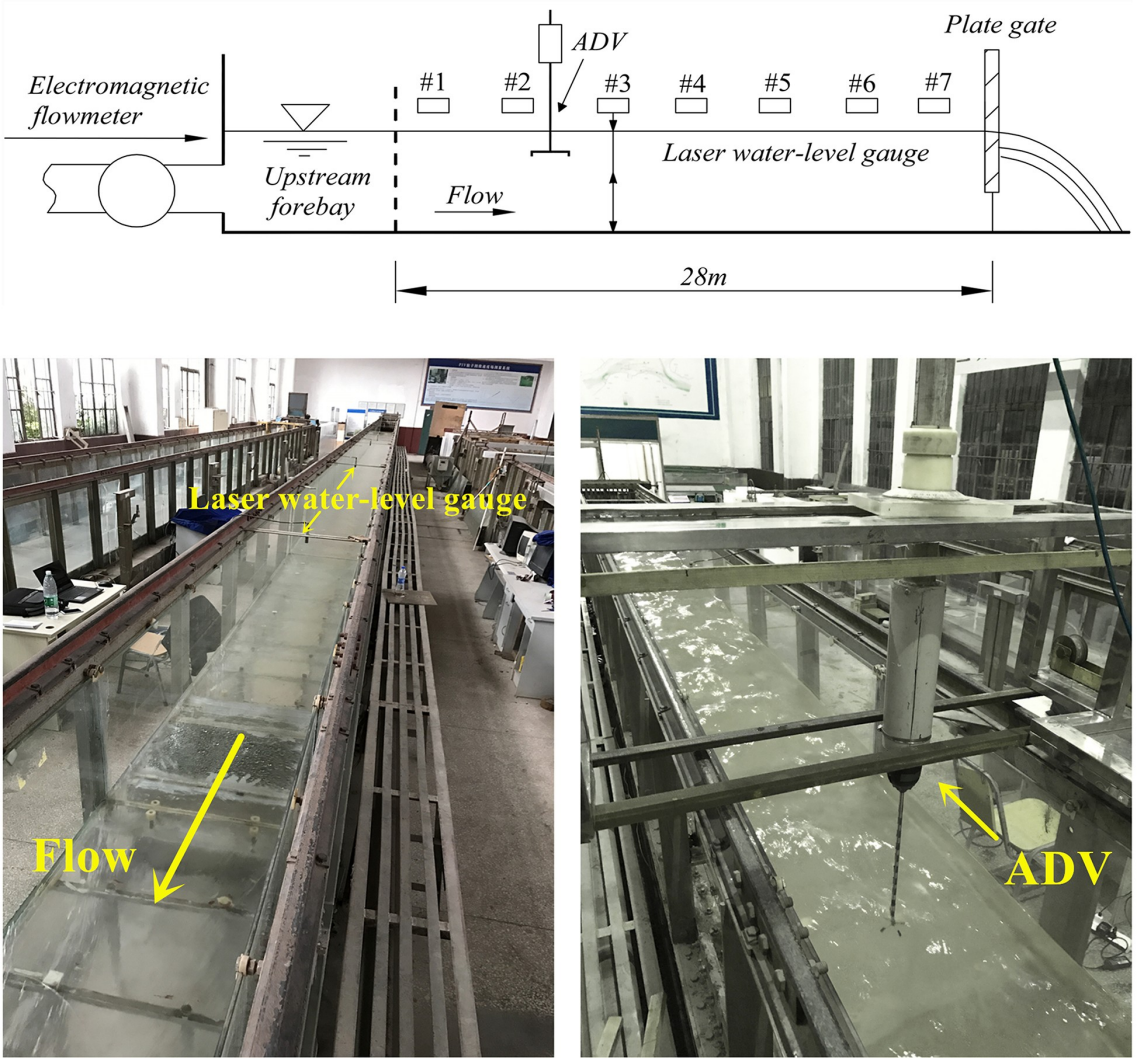
Schematic diagram of experimental set-up for open-channel flow and measuring instrument.

In the experiment, the channel bottom slope *i*_0_ is taken to be 0.002, and the intake flow *Q* is 0.06 m^3^/s. The purpose of this experiment is to simultaneously measure the water-level and the vertical distribution of the longitudinal velocity when the open-channel flow steadily develops to a uniform flow (the normal depth is *h*_0_) from a nonuniform flow. In the experiment, seven gauging points (#1-#7) are set up longitudinally (point #1 is 2 m away from the intake, and the other points are at 4 m intervals) and four velocity measuring cross-sections are at points #2, #3, #5, and #6. See Fig 4 below for the plan.

We can get a water-level lowering flow under the conditions mentioned above, and the experiment date will be applied to test the numerical model.

### Validation of numerical model

**Fig 5:**
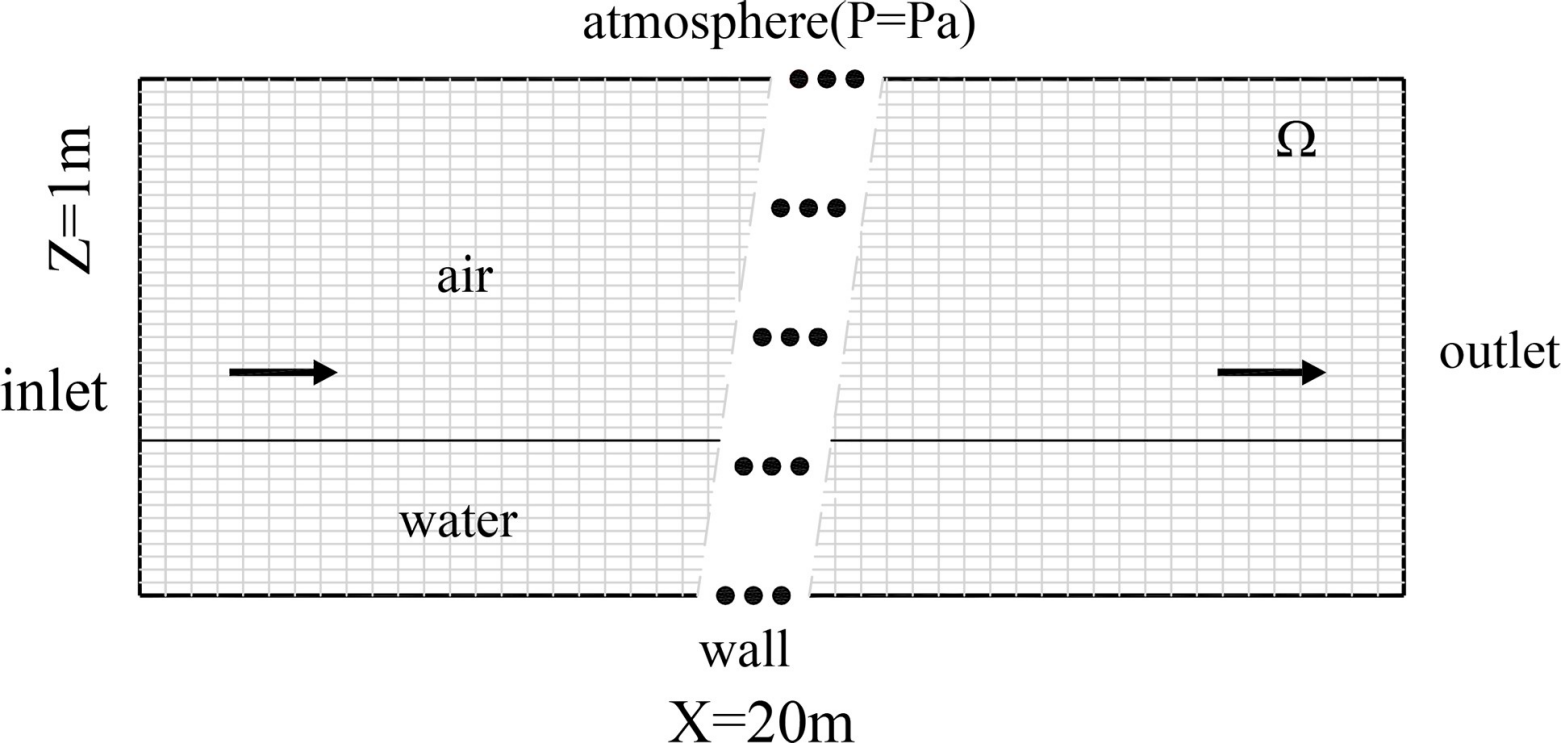
Mesh grid generation of the calculation zone. The total number of grids is 400*40, where Δ*x* = 0.05m, Δ*z* = 0.025m.

The rectangular grids are applied to the mesh generation of calculation zones, as shown in Fig 5. Forty grids and 400 grids are distributed vertically and longitudinally, respectively. The aforementioned two-dimensional numerical model of Level Set-based open-channel turbulence is applied to simulate an open-channel flow under certain experimental conditions.

In this numerical computation, the total calculation time depends on the time required for the flow reaches steady state. We employ the following criteria to determine whether the flow has reached steady state: (1) There is an obvious uniform flow section existing downstream of the open-channel flow; (2) The variation value of the length of uniform flow section in adjacent interval times is less than 5×10^−3^. In each time step, the calculation converges when the average residual error (the arithmetic mean value of the sum of all-unit residual errors) is less than 10^−3^. The value of time step is Δ*t* = 0.02s, which is associated with Courant-Friedrichs-Lewy (CFL) number less than 1.

The following sections will provide a description of the computational validation.

#### Water surface profile

**Fig 6:**
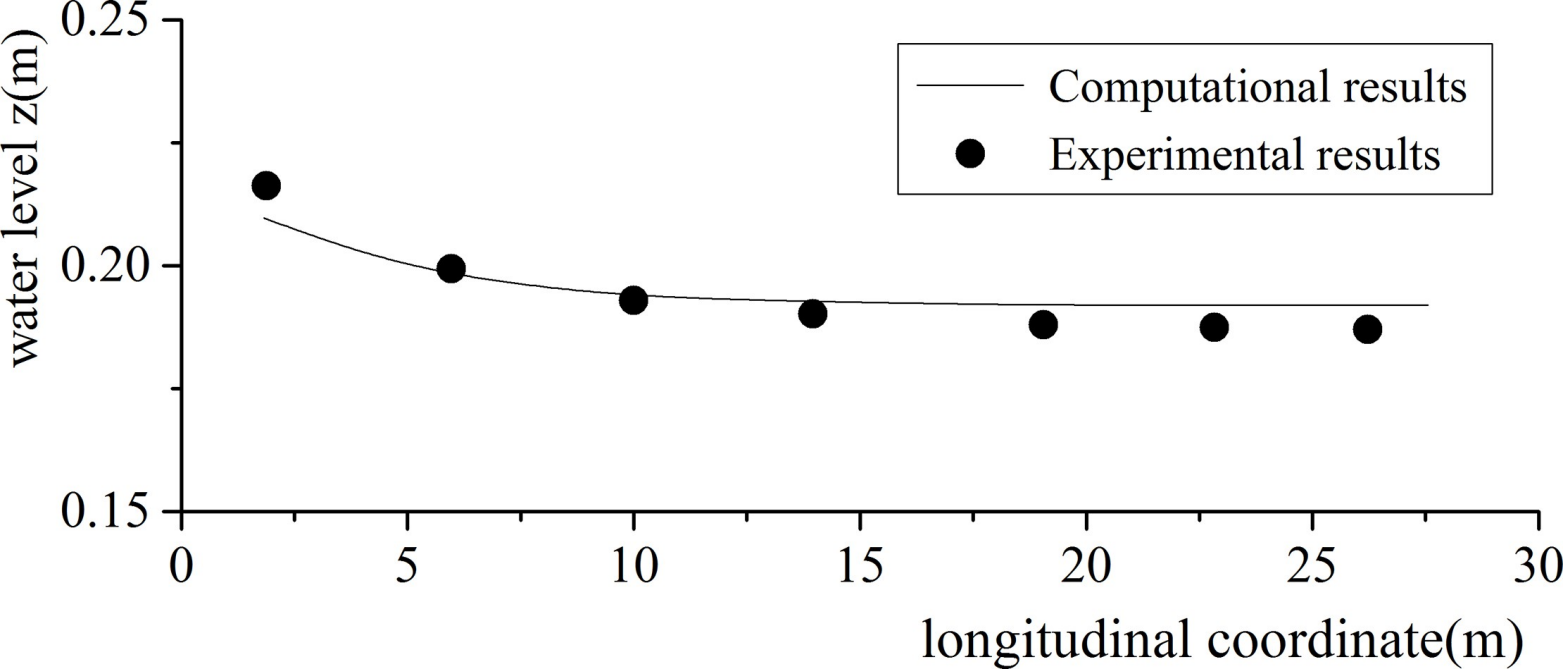
Water surface profile experiment and verification calculation.

The water surface is the water-air interface line at different locations in the longitudinal direction. Improving water surface capture precision is the purpose of the Level Set calculation in this paper. Fig 6 provides the water surface experiment and the verifying calculation results. They are well matched, as shown in the figure below.

#### Flow velocity

**Fig 7:**
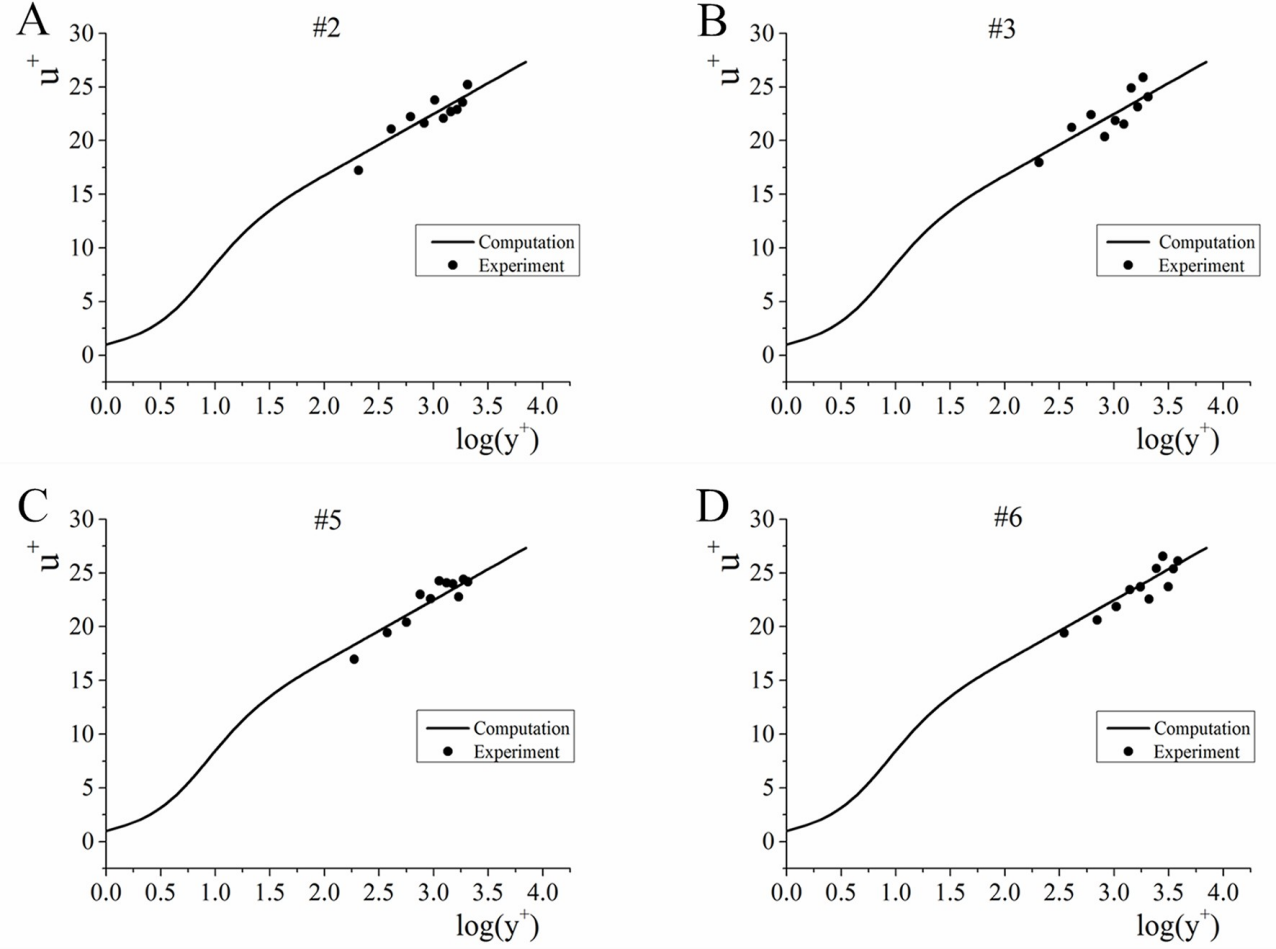
Longitudinal velocity experiment and verification calculation. A-D correspond to points #2, #3, #5, and #6 in the water channel, respectively. When the water flow stabilizes, points #2 and #3 will be in nonuniform flow area, and #5 and #6 will be in the uniform flow area.

Fig 7 below provides the longitudinal flow experiment and verifying calculation results. The *y*-coordinate is $u^{+}=\frac{u}{u_{*}}$ and the *x*-coordinate is $y^{+}=\frac{u_{*}y}{\nu }$ in the figure. The following conclusions can be determined from the figure: (1) the longitudinal flow velocity experiment and verifying calculation results are well matched, and (2) the longitudinal velocity is still subject to semilogarithmic distribution within vertical range *y*^+^>30 from the wall during the evolution of open-channel flow from nonuniform flow to uniform flow.

#### Efficiency of computation

In this numerical work, the factors that affect the computational efficiency are as follows:

1. The reinitialization of Level Set equation. The solution of Level Set equation is the key to this numerical computation, and it is also a challenging work because the Level Set function *φ*(*x*, *y*,) is implicit. And the reinitialization of Level Set equation is an important step in the process of solving. Because of the numerical effect, after the accumulation of several time steps, Level Set function no longer maintain the symbol distance characteristics in calculation. Therefore, the Level Set function must be reinitialized after each time step, which is the main reason for the heavy calculation workload. It can be argued that the reinitialization is directly related to computational efficiency.
2. The time required for the flow to reach steady state. Although we are concerned about the characteristics of steady flow, in order to get the flow most close to the actual flow, we establish an unsteady numerical model to simulate the flow developing from the initial state to the final steady state. It is obviously that the time required for flow in different conditions to reach steady state is different, that is the total time of computation, so we can easily find that this time is another factor to affect the computational efficiency.
3. The mesh size. For the same case, a more accurate free surface can be obtained by grid refinement, but the computational effort will increase greatly, thus the computational efficiency will be reduced.

From the above analysis, we can see that there are many factors that affect the computational efficiency, but the reinitialization computation is the most important factor.

## Application

**Fig 8:**
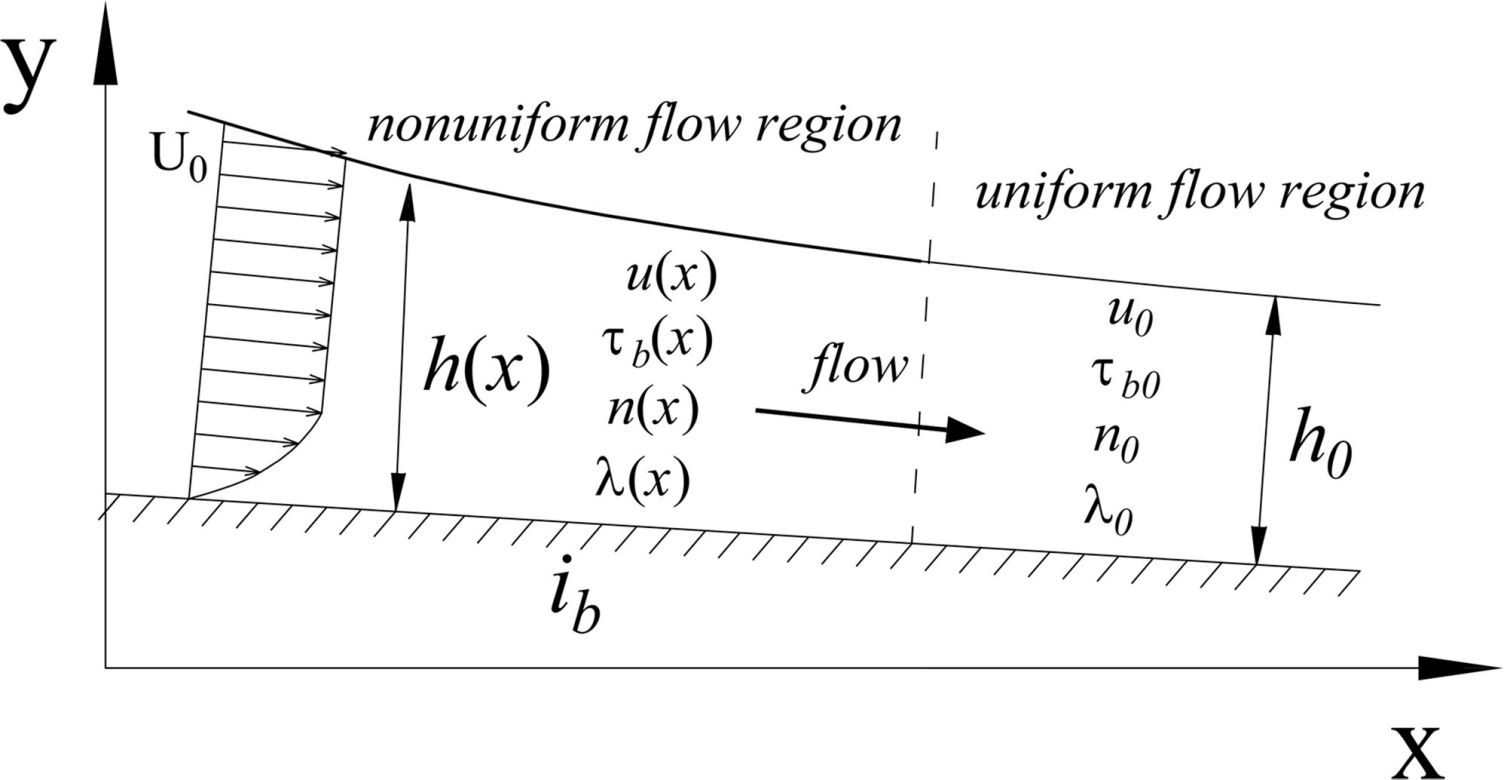
Genneral diagram of parameter variation along the flow direction. In the numerical simulation, the bottom slope *i*_*b*_ is taken as 0.002 and 0.005, and the final uniform flow Froude number *Fr*_0_ is taken as 0.1~0.8. In this section, the general features of nonuniform flow and variation features of total flow characteristic parameters are detailed on the basis of the numerical simulation results.

The same numerical model and mesh grids as the verification calculation are applied to simulate an open-channel flow in the aforesaid experimental water tank, in order to have a better understanding of general characteristics (including the water depth *h*, depth-averaged velocity *U*, and wall shear stress *τ*_b_) and variation in the flow evolution from nonuniform flow to uniform flow and to acquire the variation features of general flow characteristic parameters (energy loss coefficient *λ* and roughness coefficient *n*) under nonuniform flow. The generalization of our research content is shown in Fig 8.

### Variation of water depth and depth-averaged velocity

**Fig 9:**
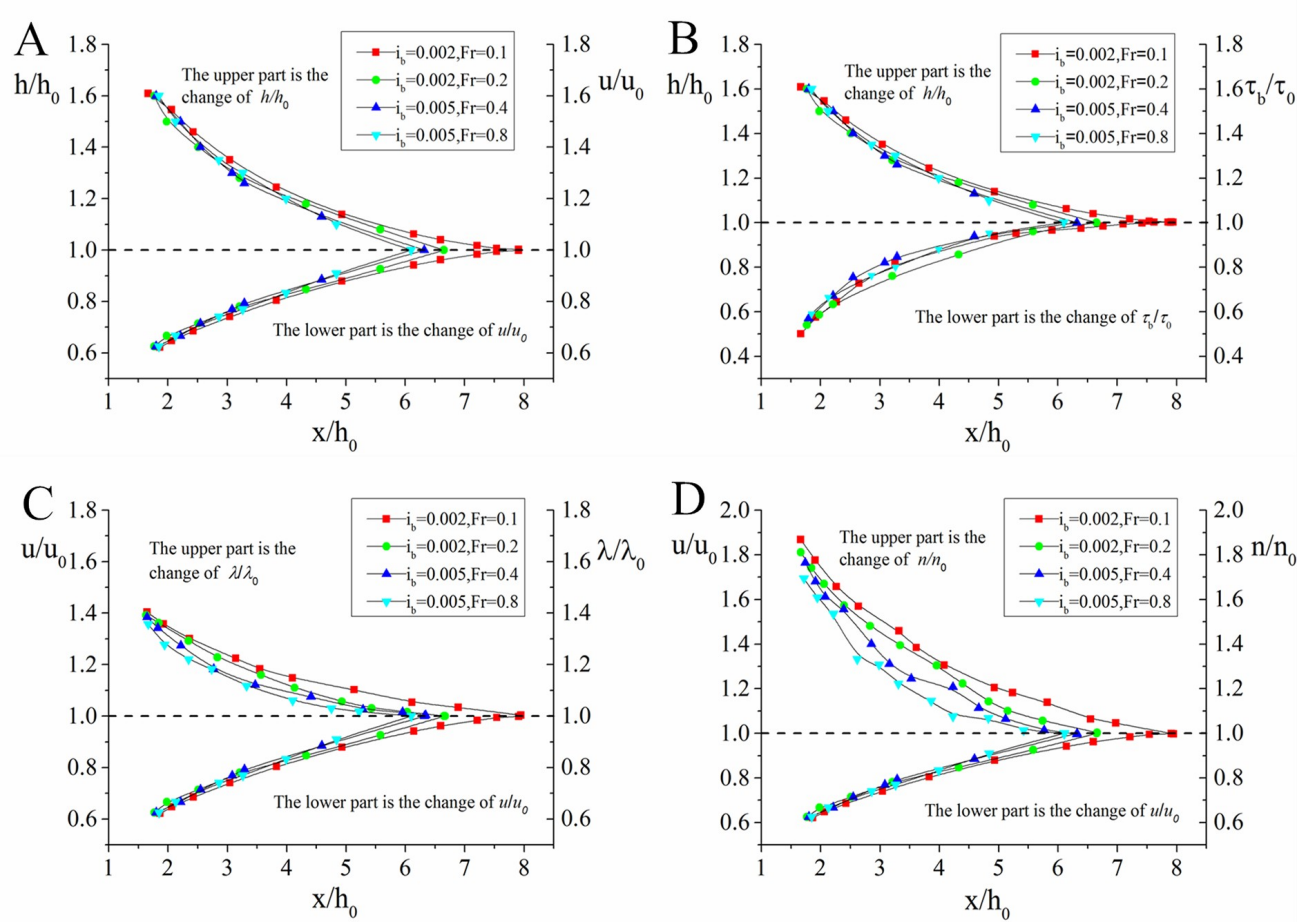
Evolution of open-channel flow uniformity. *h*_*0*_ is the normal depth of open channel flow and is used to nondimensionalize the x-coordinate. Therefore, *x/h*_*0*_ (x-axis) shows the longitudinal distance when the open-channel flow develops from nonuniform to uniform. The double y-axis (*u*/*u*_0_, *h*/*h*_0_, *n*/*n*_0_, etc.) shows the uniformity of open-channel flow.

The water depth and depth-averaged velocity when the open-channel flow is finally in the uniform state are expressed as *h*_0_ and *U*_0_, respectively, and *h*_0_ is used to nondimensionalize the *x*-coordinate. Fig 9A shows the *h*/*h*0—*x*/*h*_0_ and *U*/*U*0—*x*/*h*_0_ numerical simulation results. The following conclusions can be obtained from the figure: (1) under simulated working conditions, the water depth and depth-averaged velocity of nonuniform open-channel flow approximately change to the corresponding value of uniform flow at *x*/*h*_0_ = 6~8 of longitudinal location and (2) under the same bottom slope conditions, the higher the Froude number is, the shorter the longitudinal length is required, which is the length required for nonuniform flow reach to uniform state.

### Variation of wall shear stress

Fig 9B provides the wall shear stress *τ*_b_ process variation numerical simulation results. *τ*_b_ in the figure has been nondimensionalized by the final uniform flow wall shear stress *τ*_b0_. The following can be concluded from the figure: (1) for an open-channel flow with water-level lowering, as shown in Fig 8, when the flow tends to be uniform, wall shear stress rises along the flowing path, and wall shear stress change amplitude *τ*_b/_*τ*_b0_ is the magnitude of 0.5~1 while water depth change amplitude *h*/*h*_0_ is 1~1.6 and (2) under the same bottom slope conditions, the higher the Froude number is, the shorter the longitudinal flow length is required.

### Variation of energy loss coefficient

The energy equation of open-channel steady flow is

$$z_{1}+\frac{\alpha_{1}U_{1}^{2}}{2g}=z_{2}+\frac{\alpha_{2}U_{2}^{2}}{2g}+h_{w}$$ (18)

In Eq (18), *h*_*w*_ is the energy loss between two sections and may be calculated using the Darcy-Weisbach formula $\frac{h_{w}}{L}=\lambda \frac{U^{2}}{8gh}$. Fig 9C shows the numerical calculation results of the energy loss coefficient *λ* process variation. The following can be concluded from the figure: (1) *λ* decreases along the flowing path amid amid the transition of flow from uniform to nonuniform; i.e., the nonuniform flow energy loss coefficient is higher than the uniform flow coefficient under corresponding conditions. From the quantitative point of view, the energy loss coefficient change amplitude *λ*/*λ*_0_ varies by the magnitude of 1~1.4 while the water depth change amplitude *h*/*h*_0_ is 1~1.6 and (2) under the same bottom slope conditions, the higher the Froude number is, the shorter the longitudinal flow length is required.

### Variation of roughness coefficient

Roughness coefficient *n* is an important characteristic parameter for open-channel flow, and its relationship with the energy loss coefficient *λ* is expressed by the equation below:

$$n=\sqrt{\frac{\lambda }{8g}}h^{1/6}$$ (19)

Fig 9D provides the numerical simulation results of roughness coefficient *n* process variation. The following can be determined from the figure: (1) *n* decreases along the flowing path, i.e., the roughness coefficient of open-channel nonuniform flow is higher than that of uniform flow under corresponding conditions. Quantitatively, the roughness coefficient change amplitude *n*/*n*_0_ varies by the magnitude of 1~1.9 while water depth change amplitude *h*/*h*_0_ is 1~1.6. (2) under the same bottom slope conditions, the higher Froude number is, the shorter the longitudinal flow length required.

## Conclusion

Level Set method is an excellent free surface capturing method, but the research on it has not been widely carried out. So we established the vertical two-dimensional numerical based on Level Set method. In order to test the validation of the numerical model, the open-channel flow experiment was conducted in a long straight tank by measuring the water-level and flow velocity synchronously, and the experimental results were employed in the verification computation. Finally, the numerical computation was carried out to study the flow characteristics of open-channel flow under different flow conditions.

Based on experimental research and numerical calculation, the following conclusions are made in this paper:

1. The results of verification computation show that it is possible to precisely capture water surface profile of nonuniform flow by applying Level Set method, and the vertical distribution of the longitudinal velocity can agree with the experimental results well.
2. A semilogarithmic zone of vertical distribution of longitudinal velocity exists amid the transition of flow from nonuniform state to uniform state;
3. For an open-channel flow with a water-level lowering curve, the depth-averaged velocity and wall shear stress increase along the flowing path, but the energy loss coefficient and roughness coefficient decrease by a big margin.

## Supporting information

S1 Appendix Discretization. The discretization of motion equation in our work are outlined.
(DOCX)

S2 Appendix Source code. The kernel code of the level set method are shown in this part.
(DOCX)

S3 Appendix Reinitialization. The method how to reinitialize the level set function are given in the text.
(DOCX)

S1 Table Notation. The table shows the symbols used in this paper.
(DOCX)
