# Supplementary material for: Level Set method-based two-dimensional numerical model for simulation of nonuniform open-channel flow
Source: PLoS One. 2019 Sep 26;14(9):e0223167. doi: 10.1371/journal.pone.0223167 (PMC6762176; doi:10.1371/journal.pone.0223167)
Supplement: S1 Appendix — The discretization of motion equation in our work are outlined. (DOCX) [file pone.0223167.s001.docx]

**Discretization**

We have got the general equation for *k*, *ε* and motion equations as follow

|  | $\frac{\partial\Phi}{\partial t}+u\frac{\partial\Phi}{\partial x}+v\frac{\partial\Phi}{\partial y}=\frac{\partial}{\partial x}\left(\Gamma\frac{\partial\Phi}{\partial x}\right)+\frac{\partial}{\partial y}\left(\Gamma\frac{\partial\Phi}{\partial y}\right)\text{＋}S_{0}\left(\Phi\right)$ | (1) |
| --- | --- | --- |

where *Φ* is the generalized variable, *Γ* is the generalized diffusive coefficient, and S_0_(Φ) is the source term. These variables have different meanings in different equations.

(1) Discretization of the k,e and motion equations

We apply the FVM(the finite volume method) to discretize the general equation(1). The following discrete equation can be obtained from the integration of equation (1) over a control volume:

|  | $a_{P}\Phi_{P}=a_{W}\Phi_{W}+a_{E}\Phi_{E}+a_{S}\Phi_{S}+a_{N}\Phi_{N}+b$ | (2) |
| --- | --- | --- |

where

|  | $a_{W}=\frac{\Gamma}{\left(\Delta x\right)_{w}}\Delta y+\max\left(0,u_{w}\Delta y\right)$ | (3) |
| --- | --- | --- |
|  | $a_{E}=\frac{\Gamma}{\left(\Delta x\right)_{e}}\Delta y+\max\left(0,-u_{e}\Delta y\right)$ |
|  | $a_{S}=\frac{\Gamma}{\left(\Delta y\right)_{s}}\Delta x+\max\left(0,u_{s}\Delta x\right)$ |
|  | $a_{N}=\frac{\Gamma}{\left(\Delta y\right)_{n}}\Delta x+\max\left(0,-u_{n}\Delta x\right)$ |
|  | $a_{P}=a_{W}+a_{E}+a_{S}+a_{N}+\frac{\Delta x\Delta y}{\Delta t}$ |

For the motion equation in *x* direction, we have

|  | $\varphi=u$ | (4) |
| --- | --- | --- |
|  | $\Gamma=\nu_{T}$ |
|  | $b=-\frac{p_{e}-p_{w}}{\rho}\Delta y+\frac{\Delta x\Delta y}{\Delta t}u^{0}$ |

For the motion equation in *y* direction

|  | $\varphi=v$ | (5) |
| --- | --- | --- |
|  | $\Gamma=\nu_{T}$ |
|  | $b=-\frac{p_{n}-p_{s}}{\rho}\Delta x+\frac{\Delta x\Delta y}{\Delta t}v^{0}$ |

For the *k* equation

|  |  | (6) |
| --- | --- | --- |
|  | $\Gamma=\alpha_{k}\nu_{T}$ |
|  | $b=\frac{\Delta x\Delta y}{\Delta t}k^{0}+b^{k}$ |

where $b^{k}$is the source term consist of$G_{k}-\varepsilon$

For the e equation

|  | $\varphi=\varepsilon$ | (7) |
| --- | --- | --- |
|  | $\Gamma=\alpha_{\varepsilon}\nu_{T}$ |
|  | $b=\frac{\Delta x\Delta y}{\Delta t}\varepsilon+b^{\varepsilon}$ |

where $b^{\varepsilon}$is the source term consist of $\frac{C_{1\varepsilon}^{*}\varepsilon}{k}G_{k}-C_{2\varepsilon}^{*}\frac{\varepsilon^{2}}{k}$.

(2) Discretization of the continuity equation

Continuity equation

|  | $\frac{\partial u}{\partial x}+\frac{\partial v}{\partial y}=0$ | (8) |
| --- | --- | --- |

We use the SIMPLE algorithm to solve the pressure-velocity coupling problem. SIMPLE means Semi-Implicit Method for Pressure-Linked Equation.

The velocity at the interface of control volume is

|  | $u_{w}=u_{w}^{*}-\left(\frac{\Delta y}{a_{P}^{u}}\right)_{w}\left(\frac{p_{P}-p_{W}}{\rho}\right)$ | (9) |
| --- | --- | --- |
|  | $u_{e}=u_{e}^{*}-\left(\frac{\Delta y}{a_{P}^{u}}\right)_{e}\left(\frac{p_{E}-p_{P}}{\rho}\right)$ |
|  | $v_{s}=u_{s}^{*}-\left(\frac{\Delta x}{a_{P}^{u}}\right)_{s}\left(\frac{p_{P}-p_{S}}{\rho}\right)$ |
|  | $v_{n}=u_{n}^{*}-\left(\frac{\Delta x}{a_{P}^{u}}\right)_{n}\left(\frac{p_{N}-p_{P}}{\rho}\right)$ |

The velocity correction at the interface is

|  | $u'_{w}=-\left(\frac{\Delta y}{a_{P}^{u}}\right)_{w}\left(\frac{p'_{P}-p'_{W}}{\rho}\right)$ | (10) |
| --- | --- | --- |
|  | $u'_{e}=-\left(\frac{\Delta y}{a_{P}^{u}}\right)_{e}\left(\frac{p'_{E}-p'_{P}}{\rho}\right)$ |
|  | $v'_{s}=-\left(\frac{\Delta x}{a_{P}^{u}}\right)_{s}\left(\frac{p'_{P}-p'_{S}}{\rho}\right)$ |
|  | $v'_{n}=-\left(\frac{\Delta x}{a_{P}^{u}}\right)_{n}\left(\frac{p'_{N}-p'_{P}}{\rho}\right)$ |

where $a_{P}^{u}$ is the main diagonal coefficient of momentum equation; the velocity with subscript * is obtained from solving momentum equation; the velocity with subscript ′ is the velocity correction.

We substitute the velocity terms above into the equation (8) and then obtain

|  | $a_{P}p'_{P}=a_{W}p'_{W}+a_{E}p'_{E}+a_{S}p'_{S}+a_{N}p'_{N}+b$ | (11) |
| --- | --- | --- |

where

|  | $a_{W}=\left(\frac{\Delta y}{a_{P}^{u}}\right)_{w}$ | (12) |
| --- | --- | --- |
|  | $a_{E}=\left(\frac{\Delta y}{a_{P}^{u}}\right)_{e}$ |
|  | $a_{S}=\left(\frac{\Delta x}{a_{P}^{u}}\right)_{s}$ |
|  | $a_{N}=\left(\frac{\Delta x}{a_{P}^{u}}\right)_{n}$ |
|  | $a_{P}=a_{W}+a_{E}+a_{S}+a_{N}+\frac{\Delta x\Delta y}{\Delta t}$ |
|  | $b=\left(u_{w}^{*}-u_{e}^{*}\right)\Delta y+\left(v_{s}^{*}-v_{n}^{*}\right)\Delta x+\left(w_{b}^{*}-w_{t}^{*}\right)\Delta x\Delta y$ |

After we got the pressure correction, we can correct the pressure and velocity and get the solution as follows:

|  | $p_{P}\text{＝}p_{P}^{*}+\alpha_{P}p_{P}^{'}$ | (13) |
| --- | --- | --- |
|  | $u_{P}\text{＝}u_{P}^{*}-\left(\frac{\Delta y}{a_{P}^{u}}\right)\left(\frac{p'_{e}-p'_{w}}{\rho}\right)$ |
|  | $v_{P}\text{＝}v_{P}^{*}-\left(\frac{\Delta x}{a_{P}^{u}}\right)\left(\frac{p'_{n}-p'_{s}}{\rho}\right)$ |
