# Supplementary material for: Level Set method-based two-dimensional numerical model for simulation of nonuniform open-channel flow
Source: PLoS One. 2019 Sep 26;14(9):e0223167. doi: 10.1371/journal.pone.0223167 (PMC6762176; doi:10.1371/journal.pone.0223167)
Supplement: S2 Appendix — The kernel code of the level set method are shown in this part. (DOCX) [file pone.0223167.s002.docx]

C LevelSET

C

SBUROUTINE levelset

$INCLUDE:’SIMPLE.INC’

implicit double precision (a-h,o-z)

dimension x(0:nx+1),y(0:ny+1),fi0(0:nx+1,0:ny+1)

dimension fi1(0:nx+1,0:ny+1),fi2(0:nx+1,0:ny+1)

dimension fi3(0:nx+1,0:ny+1),right1(0:nx+1,0:ny+1)

dimension fi4(0:nx+1,0:ny+1),slope(0:nx+1,0:ny+1)

dimension u(0:nx+1,0:ny+1,2) !vel_x and vel_y

call solvefi(fi0,fi1,u,slope,x,y,nx,ny,dx,dy,dt)

call re_init(fi0,fi1,fi2,fi3,fi4,right1,x,y,nx,ny,dx,dy)

c boundary

subroutine boundary(fi,nx,ny,x,y)

implicit double precision (a-h,o-z)

dimension fi(0:nx+1,0:ny+1),x(0:nx+1),y(0:ny+1)

do 90 i=3,nx-2

do 90 j=0,2

fi(i,j)=fi(i,3)+(fi(i,4)-fi(i,3))*(y(j)-y(3))/(y(4)-y(3))

fi(i,ny+1-j)=fi(i,ny-2)+(fi(i,ny-3)-fi(i,ny-2))

& *(y(ny+1-j)-y(ny-2))/(y(ny-3)-y(ny-2))

90 continue

do 91 j=0,ny+1

do 91 i=0,2

fi(i,j)=fi(3,j)+(fi(4,j)-fi(3,j))*(x(i)-x(3))/(x(4)-x(3))

fi(nx+1-i,j)=fi(nx-2,j)+(fi(nx-3,j)-fi(nx-2,j))

& *(x(nx+1-i)-x(nx-2))/(x(nx-3)-x(nx-2))

91 continue

end

C solve level set eqation with 5th-WENO

subroutine solvefi(fi0,fi1,fi2,u,x,right1,nx,dx,dt,itrack,t)

implicit double precision (a-h,o-z)

double precision u(0:2,0:nx+1,3),right1(0:nx+1),x(0:nx+1)

double precision fi0(0:nx+1),fi1(0:nx+1),fi2(0:nx+1)

call getfiright(fi0,u,nx,right1,dx)

do 1 i=3,nx-3

fi1(i)=fi0(i)+dt*right1(i)

1 continue

call getfiright(fi1,u,nx,right1,dx)

do 2 i=1,nx

fi2(i)=0.75*fi0(i)+0.25*(fi1(i)+dt*right1(i))

2 continue

call getfiright(fi2,u,nx,right1,dx)

do 3 i=3,nx-3

fi0(i)=1.d0/3.*fi0(i)+2.d0/3.*(fi2(i)+dt*right1(i))

3 continue

itrack=0

do i=3,nx-3

if(fi0(i-1)*fi0(i).le.0) then

xx=x(i)-fi0(i)*dx/(fi0(i)-fi0(i-1))

itrack=i-1 !interface at i-1/2

endif

enddo

do i=0,nx+1

fi0(i)=x(i)-xx

enddo

write(6,*) t,xx

end

subroutine getfiright(fi,u,nx,right1,dx)

implicit double precision (a-h,o-z)

double precision u(0:2,0:nx+1,3),fi(0:nx+1),right1(0:nx+1)

do i=3,nx-3

vel=u(0,i,2)/u(0,i,1)

if(vel.gt.0) then

v1=(fi(i-2)-fi(i-3))/dx

v2=(fi(i-1)-fi(i-2))/dx

v3=(fi(i )-fi(i-1))/dx

v4=(fi(i+1)-fi(i ))/dx

v5=(fi(i+2)-fi(i+1))/dx

else

v1=(fi(i+3)-fi(i+2))/dx

v2=(fi(i+2)-fi(i+1))/dx

v3=(fi(i+1)-fi(i ))/dx

v4=(fi(i )-fi(i-1))/dx

v5=(fi(i-1)-fi(i-2))/dx

endif

t1=v1-2.*v2+v3

t2=v1-4.*v2+3.*v3

s1=13./12.*t1*t1+0.25*t2*t2

t1=v2-2.*v3+v4

t2=v2-v4

s2=13./12.*t1*t1+0.25*t2*t2

t1=v3-2.*v4+v5

t2=3.*v3-4.*v4+v5

s3=13./12.*t1*t1+0.25*t2*t2

epslon=1.e-7

a1=0.1/(epslon+s1)**2

a2=0.6/(epslon+s2)**2

a3=0.3/(epslon+s3)**2

w1=a1/(a1+a2+a3)

w2=a2/(a1+a2+a3)

w3=a3/(a1+a2+a3)

t1=v1/3.-7.*v2/6.+11.*v3/6.

t2=-v2/6.+5.*v3/6.+v4/3.

t3=v3/3.+5.*v4/6.-v5/6.

fi_x=w1*t1+w2*t2+w3*t3

right1(i)=-vel*fi_x

enddo

end

C output x,u

subroutine output(x,u,nx,gama)

implicit double precision (a-h,o-z)

double precision x(0:nx+1),u(0:2,0:nx+1,3)

open(unit=1,file='u.plt',status='unknown')

open(unit=2,file='v.plt',status='unknown')

open(unit=3,file='pressure.plt',status='unknown')

do i=1,nx

p=(gama-1)*(u(0,i,3)-0.5*u(0,i,2)**2/u(0,i,1))

write(1,101) x(i),u(0,i,1)

write(2,101) x(i),u(0,i,2)/u(0,i,1)

write(3,101) x(i),p

enddo

101 format(1x,2e20.10)

end
