# Supplementary material for: Level Set method-based two-dimensional numerical model for simulation of nonuniform open-channel flow
Source: PLoS One. 2019 Sep 26;14(9):e0223167. doi: 10.1371/journal.pone.0223167 (PMC6762176; doi:10.1371/journal.pone.0223167)
Supplement: S3 Appendix — The method how to reinitialize the level set function are given in the text. (DOCX) [file pone.0223167.s003.docx]

**Reinitialization**

After a certain time increment, the level set function *ϕ*(*x*, *y,*) no longer maintain the symbol distance characteristics in calculation. So it is necessary to reinitialize the *ϕ*(*x*, *y*) at each time-step.

Let's assume that at the t-moment, we got the level set function *ϕ_0_*. The following conditions are required to reinitialize the *ϕ*(*x*, *y, t*):

|  |  | (1) |
| --- | --- | --- |

where *S*(*ϕ_0_*) is the symbol function, equal to *sign*(*ϕ_0_*).

For ease of caculation , we rewrite the equation above as

|  |  | (2) |
| --- | --- | --- |

We smooth the sign(*ϕ*) as follow

|  |  | (3) |
| --- | --- | --- |

where *ε_2_* is a small number and it used to avoid zero denominator.

We define *s* as

|  |  | (4) |
| --- | --- | --- |

So we can obtain the *ϕ_x_* as follow

|  |  | (5) |
| --- | --- | --- |

where , is obtained from discretizing *ϕ* by fifth-order WENO scheme. It is obviously that we can get the *ϕ_y_* in the same way.

The can be discretized in third-order Runge-Kutta scheme. By now we get the new *ϕ*(*x*, *y*) at t-moment, in other words, the reinitialization of the level set function *ϕ*(*x*, *y*) is completed.
