# Supplementary material for: Level Set method-based two-dimensional numerical model for simulation of nonuniform open-channel flow
Source: PLoS One. 2019 Sep 26;14(9):e0223167. doi: 10.1371/journal.pone.0223167 (PMC6762176; doi:10.1371/journal.pone.0223167)
Supplement: S1 Table — The table shows the symbols used in this paper. (DOCX) [file pone.0223167.s004.docx]

**Notation**

| **Symbol** | **Indicates** |
| --- | --- |
| *d(x,y)* | *distance function* |
| *ϕ*(*x*,*y*,*t*) | *Level set function* |
| *S*(*x*,*t*) | *zero isoline of ϕ* |
| *u*,*v* | *instantaneous velocity* |
| *Ω,V1,V2* | *regions of calculation* |
| *p* | *total pressure* |
| *G*_k_ | *turbulent kinetic energy* |
| *θ* | *angle between open-channel and horizontal* |
| *ρ_w_*_,_ *ρ_a_* | *density of water and air* |
| *μ_w,_ μ_a_* | *dynamic viscosity of water and air* |
| ν_t_ | *turbulent viscosity coefficient* |
| *C_μ_, σ_k_, σ_ε_, C_ε1_, C_ε2_* | *all empirical coefficients* |
| *k* | *turbulent kinetic energy* |
| *ε* | *turbulent dissipation rate* |
| *u*′,*v*′ | *fluctuating velocity components* |
| $-\rho\overline{u^{'}v^{'}}$ | *Reynolds stress* |
| *Φ* | *generalized variable* |
| Γ | *generalized diffusive coefficient* |
| *S*_0_(Φ) | *source term* |
| *L(ϕ)* | *spatial operator* |
| *ϕ± x,y* | *right and left derivative of ϕ* |
| *E* | *wall roughness parameter* |
| Δ*y_p_* | *distance of rhe near-wall node P to the solid surface* |
| *H* | *Heaviside function* |
| *ε*_1_ | *low amount rectifying parameter* |
| *ε*_0_ | *an extremely small number* |
| *Sign*(*x*) | *Symbol function* |
| *i_b_* | *bottom slope of open channel* |
| *u*_*_ | *Shear velocity* |
| *Q* | *discharge* |
| *h* | *water depth* |
| *h_0_* | *normal water depth* |
| *τ*_b_ | *wall shear stress* |
| *U* | *depth-averaged velocity* |
| *Fr* | *Froude number* |
| *n* | *roughness coefficient* |
| *n_0_* | *roughness coefficient of uniform flow* |
| *h_w_* | *energy loss between two sections* |
| *λ* | *energy loss coefficient* |
| *λ_0_* | *energy loss coefficient in uniform flow* |
| *δ* | *transitional width δ between two phases* |
